# Biocompatibility of Denture Adhesives: Effects on Oral Tissues and Inflammatory Responses—Literature Review

**DOI:** 10.3390/dj13110535

**Published:** 2025-11-14

**Authors:** Paula Aleksandra Łasica, Urszula Wnorowska, Robert Bucki, Teresa Sierpińska

**Affiliations:** 1 Department of Prosthetic Dentistry, Medical University of Białystok, Ul. M. Skłodowskiej-Curie 24A, 15-276 Białystok, Poland; teresa.sierpinska@umb.edu.pl; 2 Department of Medical Microbiology and Nanobiomedical Engineering, Medical University of Białystok, 15-222 Białystok, Poland; u.wnorowska@gmail.com (U.W.); buckirobert@gmail.com (R.B.)

**Keywords:** denture adhesives, complete dentures, cytotoxicity, inflammation, biofilm formation, geriatric patients

## Abstract

**Background:** This article describes the biocompatibility of denture adhesives, focusing on their cytotoxicity towards oral fibroblasts, their influence on biofilm formation and microbial infections, and their potential to induce inflammatory responses in oral tissues. By examining these factors, we aim to shed light on the effectiveness and safety of denture adhesives, providing clinicians with helpful advice and highlighting important topics for further research. **Methods:** A systematic literature review was conducted using the Medline (PubMed) and SCOPUS databases. The search strategy included the following MeSH terms: denture adhesives, complete dentures, cytotoxicity, inflammation, and biofilm formation. Only peer-reviewed articles were included. **Results:** Studies have shown that denture adhesives may have cytotoxic effects on oral mucosal fibroblasts, as well as on biofilm formation and adhesion. Moreover, there is still little research on the effect of denture adhesives on inflammation of the denture-bearing area and cytokine production. **Conclusions:** The obtained results highlight the need for long-term patient investigations, and thorough clinical trials are absolutely essential to evaluate the actual safety of denture adhesives. Since this aspect is still under investigation, particular focus should be placed on understanding the inflammatory reactions these compounds induce. Improving the safety profile of denture adhesives will require cooperation among manufacturers, dental practitioners, and researchers to ensure that patients are adequately informed and that product formulations are improved for biocompatibility.

## 1. Introduction

Complete dentures are still the most frequently chosen replacement for toothless patients. This is primarily due to financial considerations of older patients [1,2]. Many products are available on the market to improve the maintenance of removable prosthetic restorations. These are primarily commonly known and advertised as denture adhesives. These products have been known since the 18th century, when plant pulps were used [3,4,5,6]. The current literature indicates that denture adhesives should meet several key criteria: they must be biocompatible, non-toxic and non-irritating while also demonstrating antibacterial and antifungal activity and offering ease of application [3,7,8,9,10].

According to research, approximately 6.9% of patients using removable dentures use denture adhesives systematically [11]. The ISO (International Organization for Standardization) divides dental adhesive preparations into soluble forms (as powders, creams, sheets or tapes) and insoluble forms (pads and wafers) [12,13,14].

Due to such widespread access to denture adhesives, the question arises whether these agents are safe for our patients. According to the literature, the ingredients responsible for adhesion include pectins, gelatin, karaya gum, sodium carboxymethylcellulose and hydroxymethylcellulose. Antibacterial and antifungal agents include sodium borate, hexachlorophene, sodium tetraborate and propyl hydroxybenzoate. Antibacterial and antifungal agents include sodium borate, hexachlorophene, sodium tetraborate and propyl hydroxybenzoate. Fillers include mainly magnesium oxide [15]. The available literature from recent years clearly indicates the negative effects of continuous use of denture adhesives by prosthetic patients, often without medical indications. This underscores the need for a reliable assessment of the impact of these products on patients health.

## 2. Materials and Methods

**Figure 1:**
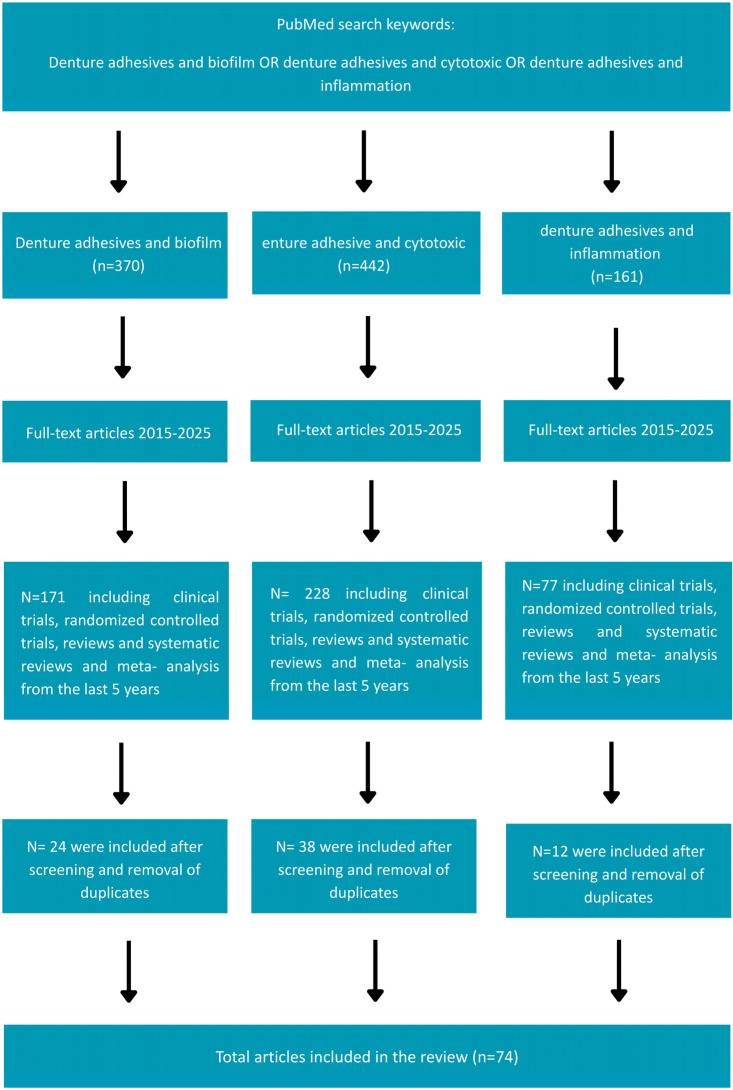
Prisma flowchart for study selection.

A systematic search of the medical literature was conducted in the Medline (PubMed) and SCOPUS databases to identify all peer-reviewed publications relevant the objectives of this review. The PRISMA guidelines were strictly followed to ensure transparency in the search strategy (Supplementary Checklist S1), inclusion/exclusion criteria, and study selection process, which are now presented in a PRISMA flowchart (Figure 1. PRISMA flowchart for study selection). The PICO framework was applied to guide the following research question: “What effect do denture adhesives have on the oral mucosa?” And it is now clearly defined in the manuscript as follows:

(P) Population: patients using removable dentures;
(I) Intervention: denture adhesives available and used by patients, and their effects on the oral mucosa, including cytotoxicity, adhesion and biofilm formation, and inflammatory responses;
(C) Comparison: healthy oral mucosa;
(O) Outcome: levels of cytotoxicity, adhesion and biofilm formation, and induction of inflammatory responses on the oral mucosa.

The inclusion criteria comprised studies describing the effects of denture adhesives on cytotoxicity, adhesion and biofilm formation, as well as inflammatory reactions. Both in vitro and clinical studies were included. Eligible study designs encompassed clinical trials, randomized controlled trials, reviews, systematic reviews, and meta-analyses. Exclusion criteria included non-peer-reviewed articles, conference abstracts, case reports, non-English publications, and studies published more than five years prior to the search date (this restriction was applied to ensure the synthesis reflects the most recent evidence base).

## 3. Results

### 3.1. Denture Adhesives: Clinical Uses and Advantages

**Figure 2:**
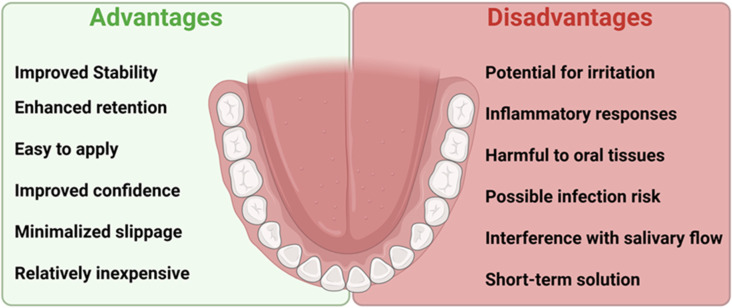
Some of the potential benefits and challenges related to the use of denture adhesives.

There are no recommendations in the literature for the use of denture adhesives as a permanent, necessary element of the use of complete dentures. Recommended situations include immediate dentures, those with significant resorption of the alveolar processes, neuromuscular control disorders, xerostomia, or significant facial defects caused by cancer or trauma. Additionally, denture adhesives can be used to improve the mental comfort of athletes, artists, and TV personalities, because according to research, the strength of maintaining the complete denture on the base after using the adhesive is more than twice as high [16,17]. Denture adhesives help keep dentures in place and improve comfort, but prolonged use can encourage microbial growth, which may lead to inflammation of the mucous membrane as well as bacterial and fungal infections (Figure 2. Some of the potential benefits and challenges related to the use of denture adhesives).

Research has shown that various microorganisms can thrive in these adhesives, potentially affecting both oral and overall health.

### 3.2. Microbial Risks Associated with Adhesive Use

As seen in Table 1, *Candida albicans* is the most commonly reported fungus, often causing prosthetic stomatitis, an inflammatory condition of the oral mucosa, and, in severe cases, even systemic infections [18,19].

Additionally, bacteria like *Staphylococcus*, *Streptococcus*, and *Fusobacterium* have been found in denture adhesives, which can lead to infections and bad breath [20,21].

Xerostomia may raise conflicting feelings regarding the recommendations for the use of these products, because dry mouth may cause allergic reactions and bacterial and fungal contamination [22,23].

Many publications focus on the positive aspects of the use of adhesive preparations, pointing to a significant improvement in the stabilization and retention of complete dentures and thus a positive impact on the quality of life and satisfaction of edentulous patients [4,24,25,26,27,28,29,30,31,32]. Moreover, the use of adhesives reduces the risk of irritation of the prosthetic base, improves chewing efficiency, improves the quality of pronunciation and accelerates adaptation immediately after receiving a new set of dentures [33,34,35,36,37,38].

Publications from around the world report various negative effects of denture adhesives on both oral and systemic health. The reports indicate primarily the cytotoxic effect of these materials on fibroblasts of the oral mucosa. This may be due to the largest number of studies on this topic. Additionally, there are studies showing the harmful impact of adhesive agents on the formation of biofilm and bacterial and fungal infections. We know the least about its impact on the creation of inflammatory reactions, but we know that it has a negative impact.

It should be emphasized that the widespread availability of adhesives without medical supervision necessitates an assessment of their impact on the oral mucosa, and consequently on the development and progression of prosthetic stomatopathy.

This work focused on the analysis of available research and the final assessment of the impact of commonly available denture adhesives on the cytotoxic effect on fibroblasts of the oral mucosa, the impact on the growth and development of biofilm and bacterial and fungal infections, and their impact on the induction of inflammatory processes in the prosthetic base.

### 3.3. Cytotoxic Effects of Denture Adhesives on Oral Fibroblasts

The use of removable prosthetic restorations is often associated with the risk of mechanical damage to the prosthetic base through permanent irritation with the denture plate, which results in erosions and in extreme cases, ulcers. This is when the fibroblasts of the oral mucosa are most exposed to the ingredients of denture adhesives.

Fibroblasts constitute the basic group of cells of the lamina propria of the oral mucosa, responsible for inflammation and regeneration of the prosthetic base [39,40]. These cells take part in various stages of the wound healing process. At first, they are responsible for the crosstalk between immune cells. Then, produce extracellular matrix (ECM) components and establish crosstalk with keratinocytes. At the very end, are responsible for matrix metalloproteinases (MMPs) and matrix component secretion [41,42,43,44,45,46,47].

Emerging reports on the cytotoxicity of denture adhesives prompted scientists to study primarily fibroblast cells. And mainly fibroblast cells obtained from the gums were used [12,48]. This is due to the fact that since the discovery of fibroblast cells in the 19th century, they have been very easy to develop in vitro as cells for medical research. And after discovery HeLa cells in 1951, they are the most frequently chosen cell line for research [49,50,51].

Numerous methods are used in research, for example, the MTT assay (colorimetric assay for assessing cell metabolic activity), the flow cytometric apoptosis assay, ELISA (enzyme-linked immunosorbent assay) and XTT (Cell Viability Assay) [47,48,52,53,54].

The safety of denture adhesives largely depends on their biocompatibility. Researchers have investigated their potential toxicity using both laboratory (in vitro) and real-world (in vivo) studies. The in vitro methods described in the referenced literature included pH assessment, evaluation of cell viability, examination of live and dead cells, as well as the MTT assay (used to determine cellular viability or metabolic activity in microcapsules). The in vivo studies primarily involved the quantitative evaluation of human mucosal fibroblasts. As shown in Table 2, in vitro experiments suggest that many denture adhesives can harm fibroblasts, leading to cell death and damage to the oral mucosa [15,48]. However, findings from in vivo studies indicate that these adhesives may not have a significant long-term impact on the microbial balance in the mouth [55].

These results underscore the necessity of additional research to ascertain the long-term impacts of adhesive components on oral tissues and overall health.

In 2020 Yamada et al. and Lopez-Garcia et al. confirmed the cytotoxic effect of denture adhesives on fibroblasts of the oral mucosa. It was concluded that the cytotoxicity of adhesive materials depends on individual conditions, the type of material, and the concentration and duration of exposure. Furthermore, the variation in cytotoxicity among the tested adhesives was attributed primarily to differences in their composition. It was also confirmed that these agents induced necrosis and apoptosis of fibroblast cells of the oral mucosa [15,16]. López-García et al. specifically found that denture adhesives with zinc salts—such as Fixodent Pro Duo Protection and Fixodent Pro Plus Duo Protection—markedly reduced cell viability and caused necrosis in human gingival cells. These adhesives caused unusual cell shape and raised reactive oxygen species (ROS) synthesis. The findings implies that the observed cytotoxic effects in these adhesives could be caused by zinc content [15]. Sobolewska et al. also examined the components in several denture adhesives and found possibly harmful ones. For example, propylparaben (Propyl 4-hydroxybenzoate- antimicrobial preservative) used in COREGA Extra Strong has been linked to cytotoxic effects on human fibroblasts. Another chemical compound associated with cytotoxicity and included in Protefix^®^ is methyl benzoate (methyl benzenecarboxylate), which is commonly used as a flavoring agent and fragrance component. Moreover, some adhesives with a low pH—averaging about 5.5—may cause enamel demineralization. The study emphasizes that negative cellular effects in denture adhesives could be caused by preservatives and other components [54]. These results highlight the need to evaluate the specific components in denture adhesives, as some of them—such as zinc salts (used to improve adhesion), propylparaben (a preservative), and methyl benzoate (a flavoring and fragrance agent)—have been linked to cytotoxic effects on oral fibroblasts. Costa et al. carried out a scoping study in 2022 to evaluate denture adhesive possible toxicity. To assess the cytotoxic effects of these adhesives on oral tissues, they examined 33 papers—14 in vitro and 19 clinical studies. According to the review, most widely sold denture adhesives have a dose-dependent cytotoxic effect on fibroblasts and keratinocytes; larger concentrations cause increasing cell toxicity. The study further underlined that fibroblast regeneration is especially reduced in elderly patients, implying a higher risk of poor oral tissue healing in this population. Furthermore, several patients reported neurological or hematological changes linked to the too high use of denture adhesives. The writers underlined the need of utilizing well-adapted detachable dental prosthesis, guaranteeing appropriate patient follow-ups, and, where advised, giving precise directions for the use of denture adhesives [33].

In the available literature, we can find that specific ingredients of some denture adhesives have a particularly negative cytotoxic effect, both local and systemic. The addition of calcium and zinc (Zn) salts to denture adhesives, although it improves the effectiveness of retention, may reduce their biocompatibility. A relationship has been noted between the presence of zinc in the adhesives and high levels of zinc in blood serum (hyperzincemia) and low levels of copper in serum (hypocupremia), which leads to irreversible neurological changes [14,56]. Based on the results of the analyzed studies, it can be concluded that dental adhesives, through their cytotoxic effect, lead to a number of negative effects that should not occur when using biocompatible products. The concentration of adhesives and the time of contact with the mucous membrane are very important, which is extremely difficult to control in geriatric patients. As we know, limited manual skills, numerous diseases of elderly patients and lack of awareness of the risk mean that patients often do not follow the recommendations in the leaflet and rarely replace the dental adhesive used, or even sleep with it. Therefore, they are particularly vulnerable to the negative effects of these agents [57].

Additionally, it should be emphasized that due to the presence of numerous studies and various research methods used, it would be advisable to introduce standardization for easier analysis of the results. Standardization in the form of a clear and reliable protocol for assessing the cytotoxicity of denture adhesives on oral mucosal fibroblasts, both in vitro and in vivo, would be a considerable improvement.

### 3.4. Microbial Biofilm Formation and the Role of Denture Adhesives in Prosthetic Stomatitis

A biofilm is a highly organized structure of microorganisms intertwined in a three-dimensional extracellular matrix. In the oral cavity, adsorption of salivary membrane proteins occurs first, followed by adhesion and growth of microorganisms. Due to the numerous surfaces for adhesion of a biofilm in the oral cavity and unlimited access to nutrients, it is an extremely important etiological factor of many oral diseases and prosthetic stomatitis. The structure of the biofilm and the defense mechanisms between the microorganisms colonizing the biofilm provide numerous benefits such as protection against the effects of antibacterial and fungal agents. This constitutes a real treatment problem and indicates the need to prevent biofilm formation [58,59,60].

It is estimated that denture-related oral inflammation and damage occur in approximately 67% of complete denture users, which significantly worsens the quality of life and satisfaction with the restorations used by our prosthetic patients [61]. Both local and systemic factors can contribute to the multifactorial etiology of prosthetic stomatitis. The most include injuries caused by the denture plate, wearing dentures at night, poor denture hygiene, microorganisms infections, low salivary pH, frequent consumption of sugar and factors predisposing to systemic diseases such as aging, malnutrition, immunosuppression, radiotherapy, and antibiotic therapy [62,63]. Numerous types of microorganisms inhabit the oral cavity, including *Candida*, *Streptococcus*, *Eubacteria*, *Fusobacterium*, *Capnocytophaga*, *Eubacteria*, *Staphylococcus*, *Eikenella*, *Porphyromonas*, *Leptotrichia*, *Prevotella*, *Peptostreptococcus*, *Treponema*, and *Actinomyces*.

Excessive growth of microorganisms is closely related to excessive plaque deposits. Denture plaque forms through microorganisms colonization from saliva and whole oral mucosa. Nutrients for growth come from food, saliva, epithelial cell exfoliation and inflammatory exudate. There is a close correlation between poor oral hygiene and the biofilm and denture plaque growth. The plaque can contain many harmful products produced by both yeast and bacteria, including enzymes, toxins and antigens of microorganisms [64]. When the mucous membrane becomes inflamed its barrier function against microbiological products is much reduced. Former Bartels research indicated that *Candida albicans* metabolism products lead to inflammation after long contact with the mucous membrane [65].

The role of *Candida albicans* in the pathogenesis of prosthetic stomatitis is well known. It is known that other fungal species, such as *Candida glabrata* and *Candida tropicalis*, can also infect the surface of the denture base and, moreover, that there is a close correlation between the presence of *Candida albicans* and other pathogens in patients who do not adhere to the nighttime break in denture use. Fungal infection is the most common presentation of denture stomatitis, affecting up to 50 to 60% of denture users [66,67,68].

Jeganathan et al., based on his research, found that stomatitis occurred in 61% of patients wearing dentures at night, compared to 18% of those who did not wear their dentures at night [69]. Denture adhesives may cause greater growth not only of *Candida albicans* but also of oral bacteria. These products may play a role in halitosis by stimulating the growth of *Streptococcus oralis*, *Prevotella oralis*, *Fusobacterium nucleatum*, and *Streptococcus mutans* [21,70].

Available research on denture adhesives does not present clear results. Kim et al. in vivo studies have shown that the use of denture adhesives does not significantly change biofilm formation for a period of 14 days, and Oliveira et al. studies show no change over 60 days [71,72,73]. In contrast, Stafford et al. asserted that denture adhesives could potentially influence the growth of microorganisms, while Makihira et al. reported that denture adhesives tended to show yeast growth [74,75]. These discrepancies may arise from differences in study design, observation periods, and microbial detection methods. While the short-term studies suggest minimal or no microbial changes, longer-term or more sensitive analyses indicate that certain formulations may promote colonization, particularly by yeasts. Therefore, it has become popular among manufacturers to introduce an ingredient responsible for antifungal and antibacterial properties. Effective treatment of stomatitis involves treating not only the mucous membrane but also the denture base. Adding antimicrobial agents to denture adhesives could inhibit the colonization and growth of *Candida albicans* and other species and reduce the severity of the inflammations.

Rajaram et al. examined 3 forms of denture adhesives: powder, cream, and strips (Secure, Bioforce USA, Ghent, New York, NY, USA). The aim of the study was to test the antifungal properties of the product based on its specific form. His research concluded that the powder and cream forms had the most appropriate pH, leading to the lowest colony growth [76]. However, Leite et al. examined 30 completely edentulous denture wearers during the 15-day period. He found that the adhesives did not affect the biofilm on the palatal mucosa, but they did affect the internal surface of the dentures [77]. Taken together, these findings indicate that the impact of denture adhesives on microbial growth is context-dependent: while some clinical studies report negligible changes, others highlight specific risks such as yeast proliferation or surface-related biofilm formation. This underlines the importance of standardizing methodologies and evaluating not only the duration of adhesive use but also the composition and pH of the products tested.

The most commonly used antimicrobial agents are hexachlorophene, sodium tetra borate, methyl salicylate, and sodium borate. It is known that the long-term use of denture adhesives may affect the oral microflora by supporting the growth of some microorganisms and inhibiting others [78,79]. Some studies indicate that adhesives inhibit the growth of oral microorganisms, while others confirm their impact on the population growth of *Streptococcus mitis* and *Candida albicans* [80,81,82]. Neill et al. suggested that in the case of proper oral hygiene, adhesives did not cause any growth of oral microorganisms [83]. Ozkan et al. examining 30 patients also stated that the number of accumulated microorganisms was not significantly different than those of the denture wearers in control group [78].

Costa et al. divided the samples into four groups for testing: control (no adhesive), ultra Corega cream, Corega strip adhesive, and ultra Corega powder (GlaxoSmithKline) [84]. Fluorescence microscopy and the counting of colony-forming units checked the formation of biofilm. Costa et al. also examined the impact of hygiene on the development of pathogens: brushing with distilled water, brushing with Protex soap, brushing with Colgate toothpaste, immersion in Corega Tabs and immersion in Corega Tabs followed by brushing with the solution itself [84]. The results showed that *Candida albicans* formed more biofilm in the strip and powder form of denture adhesive, while Staphylococcus aureus also formed more biofilm in the strip. Excessive development occurred when examining all forms of the denture adhesives. Additionally, it was found that brushing with Colgate and Protex soap was most effective for removing the adhesives, which is very important as we know that this material can favor biofilm accumulation [84].

Other research was conducted by de Oliveira et al. who wanted to verify whether the Ultra Corega Cream and Corega Strip Denture Adhesive interfere with the microbial adhesion and biofilm formation by *Candida albicans* and *Lactobacillus casei* in single- and mixed-species settings, and observe whether synergistic or antagonistic relationships between these species emerge [85]. The research was conducted in 3 product groups: without adhesive (control group), with ultra Corega Cream adhesive and Corega Strips adhesive. These groups were divided into three subgroups: *Candida albicans*, *Lactobacillus casei and Candida albicans* with *Lactobacillus casei* (mixed-species) [85]. As a test result, de Oliveira et al. stated that both of the denture adhesives increased the adhesion of *Candida albicans* but not the *Lactobacillus casei*. Additionally, it was noticed that synergism or antagonism was not observed between the two microorganisms [85].

There are no studies that would directly indicate which adhesive component influences greater adhesion of microorganisms. It can be assumed that those responsible for the adhesion of adhesives to the prosthetic base. However, it is possible to take a closer look at research into additional ingredients that would have antimicrobial properties.

Chung et al. investigated the antimicrobial and antibiofilm activity of two copper complexes derived from Schiff base. Research concerned *Staphylococcus aureus*. Copper has been found to be able to, among other things, disrupt cell wall synthesis, inactivate cellular enzymes, denature proteins in the body, and form a hydrogen bond via the azomethine group with the cellular components. There are no reports so far about the possibility of bacteria becoming resistant to copper [20]. Pereira et al. also studied copper-based metallic compounds (phenanthroline, bipyridine, pyrazinamide, isonicotinic acid) against biofilms formation of *Staphylococcus aureus*, *Staphylococcus epidermidis*, and *Escherichia coli*. The results presented in this study showed promising antimicrobial and antibiofilm activities [86]. Oliveira et al. found that pyrazoles increase the effect of antifungal agents against candida and that pyrazole at 1560 μg (3.02 wt%) was the most efficient, at lowest cytotoxic effect [87]. Mamtora et al. confirmed the effective antifungal effect of the miconazole nitrate added to denture adhesives [88]. Peralta et al. describes the antimicrobial action of silver nanoparticles against *Candida albicans* [89].

A final assessment of the impact of adhesive agents on the growth of primarily *Candida albicans* and *Streptococcus mutans* on the oral mucosa and on the denture, base is made due to the lack of standardized tests focused on microorganisms (Table 3). This is extremely important, especially when the hygiene of geriatric patients is poor [64,90].

### 3.5. Inflammatory Response of the Oral Mucosa to Denture Adhesives

The oral mucosa’s inflammatory response is a critical indicator of oral cavity pathology. Cytokines, a diverse group of signaling proteins, are essential for the mediation of communication among cells of the cellular and humoral immune systems, as well as hematopoietic processes during inflammation. These proteins not only promote the proliferation of hematopoietic cells and other tissues through pro-inflammatory pathways and receptors, but they also activate host defense mechanisms, such as fever. The extent of their action in the body during the response suggests more than just local, but also systemic effects [91,92].

The inflammatory response of the oral mucosa serves as a critical indicator of oral health, with cytokines playing a pivotal role in mediating immune reactions. Key cytokines involved in oral inflammation include prostaglandin E2 (PGE_2_), interleukins such as IL-1, IL-6, IL-8, IL-10, and tumor necrosis factor-alpha (TNF-α). Alterations in the levels of these cytokines can signal adverse biological effects stemming from the use of denture adhesives. Notably, IL-1 and IL-6 are integral to the inflammatory process due to their wide-ranging effects, with elevated levels observed in biopsies from inflamed gingival tissues. Changes in the levels of these cytokines can serve as indicators about the unfavorable biological effects of denture adhesives [93,94]. Biopsies from inflamed gingival tissues have revealed elevated levels of these interleukins [95,96,97,98]. Studies on some universal dental adhesives have shown that exposure can cause an overproduction of inflammatory markers, hence possibly inciting inflammatory reactions upon contact with oral tissues. Specifically, all dental adhesives (bonding systems for enamel and dentin) that have been tested, demonstrated the ability to alter inflammatory patterns, resulting in an increase in IL-6 and IL-8 expression, immediately following exposure, with a decrease at 48 h. Conversely, the secretion of IL-1β into the extracellular compartment is likely responsible for the substantial increase in IL-1β levels observed at 48 h. This finding poses critical inquiries regarding the fundamental mechanisms of inflammation and establishes a basis for future research. Research indicates that the complex interactions among inflammatory pathways involve key components like NF-κB and cathepsin B, which play important roles in regulating cytokine expression [99]. Certain dentin/enamel adhesives, specifically FuturaBond M+ and Prime&Bond Active, are associated with NF-κB-mediated cytokine expression, whereas bonding agents such as Optibond Solo Plus and Universal Bond do not exhibit this connection, indicating a potential regulatory role of the cathepsin B pathway in these cases [99,100].

The immune defenses of the oral mucosa consist of both specific and non-specific mechanisms. Non-specific immunity is characterized by macrophage activation, phagocytosis, and opsonization, while specific immunity is initially dependent on salivary antibodies and is subsequently followed by cytotoxic T-cell activation [64]. Adequate saliva secretion and pH levels are essential for the preservation of biocompatible conditions in the oral cavity. The buccal mucosa is at risk of inflammatory responses due to xerostomia, a condition that frequently necessitates the use of adhesives. Love et al. conducted research that indicated that certain adhesives could reduce oral pH to levels that were detrimental to enamel health. Karaya gum was identified as a particularly deleterious component [101].

A dry mucous membrane is more prone to mechanical damage and inflammatory responses, for instance, those induced by plaque accumulation [102]. Only one in vitro study (Perin Leite et al. [98]) have investigated the impact of denture adhesives on cytokine-mediated inflammation, despite their importance in mediating pro-inflammatory processes. No in vivo studies were found. The need for further research into the effects of adhesives on diverse patient populations is underscored by the fact that most research remains to be done. Future studies should focus on well-designed, long-term clinical trials that assess not only inflammatory biomarkers but also patient-related outcomes across different age groups, systemic conditions, and durations of adhesive use. Such investigations are essential to provide evidence-based guidance for safe and effective denture adhesive application. Utilizing the ELISA test, Perin Leite et al. evaluated the release of cytokines in response to three varieties of denture adhesives—Ultra Corega cream, Corega powder Fixador Ultra, and Corega strips (GlaxoSmithKline Brasil Ltd.a)—to determine the levels of IL-1β, IL-6, and TNF-α. The study determined that denture adhesive strips did not substantially affect the release of cytokines from human oral keratinocytes. However, the cream and powder variants were able to induce cytokine release after prolonged exposure [98]. Additionally, the emission of formaldehyde, a known allergen associated with inflammation in denture wearers, was observed [103,104].

Further proving cytotoxic effects on human fibroblasts, denture adhesive creams cause cell viability declines ranging from 45.87% to 61.13% [54]. These results imply that denture adhesives could cause inflammatory reactions in the oral mucosa, thereby maybe producing negative clinical results. Thus, it is essential for next studies to concentrate on clarifying the processes by which denture adhesives affect cytokine generation and inflammatory paths. Developing safer adhesive formulations and creating rules to reduce inflammatory hazards connected with their use depend on such studies, thus they will be absolutely important.

## 4. Conclusions

Denture adhesives used unsupervised carry major hazards for the health of prosthesis patients. Usually sold straight to consumers, these products are difficult to control and could cause both local and systematic health problems.

Most research confirms that some denture adhesives might induce development of oral candidiasis, possibly leading to systemic infections, and metabolic abnormalities such as hyperzincemia and hypocalcemia. Some denture adhesives show cytotoxic effects on human fibroblasts, according in vitro studies. In vivo studies are lacking. Some scientific reports, depending on the denture adhesive investigated, provide conflicting findings regarding the level of cytotoxicity of these products, with some even indicating its absence. Furthermore, concerns regarding the long-term biocompatibility of denture adhesives arise from the fact that some formulations have been associated with mucosal irritation and inflammation, as confirmed by numerous scientific reports over the years. These findings underscore the absolute necessity of long-term clinical studies and large-scale clinical trials to properly assess the safety of denture adhesives in everyday practice. As this issue remains under investigation, particular attention should be given to understanding the inflammatory responses triggered by these compounds. Unfortunately, studies specifically addressing inflammatory reactions to denture adhesives are still lacking. Improving the safety profiles of denture adhesives will require close collaboration among manufacturers, dental practitioners, and researchers to ensure that patients are adequately informed and that product formulations are continuously refined to enhance biocompatibility.

## Figures and Tables

**Table 1:** Microorganisms associated with denture adhesives in denture wearers.

Authors, Year	Type of Study	Microorganism	Health Risks
Darwish et al., 2021 [18]	In vitro	*Candida albicans*	prosthetic stomatitis characterized by inflammation and redness of the oral mucosa
Tamura et al., 2018 [19]	In vitro	*Candida albicans*	systemic candidiasis
Chung et al., 2021 [20]	In vitro	*Staphylococcus species*	complications ranging from minor to life-threatening infections
Polyzois et al., 2013 [21]	In vitro	*Staphylococcus species*	halitosis
Polyzois et al., 2013 [21]	In vitro	*Fusobacterium*	halitosis

**Table 2:** Summary of cytotoxicity studies of denture adhesives.

Authors, Year	Type of Study	Adhesives Tested	Test Method	Cytotoxicity Outcomes
Yamada et al., 2020 [16]	In vitro	Six dentures: Faston, Poligrip Powder, New Poligrip Free, Tafugurippu Kurimu, Polident Adhesive, and Tafugurippu Tomei	examination of live and dead cells, pH assessment	cytotoxic effect of denture adhesives on fibroblasts confirmed regardless of type of material; necrosis and apoptosis of fibroblast cells of the oral mucosa
Lopez-Garcia et al., 2020 [15]	In vitro	Six denture adhesives: Poligrip Flavour Free, Fixodent Pro Duo Protection, Novafix cream, Fitty^®^ Dent, Polident Total Action, and Fixodent Pro Plus Duo Protection	MTT test	cytotoxic effect of denture adhesives on fibroblasts confirmed; necrosis and apoptosis of fibroblast cells of the oral mucosa
Karwa et al., 2017 [55]	In vivo	Three denture adhesives: Fixodent, Ultradent, and Polygrip	quantitative methods	The tested adhesives did not significantly change the denture microbiome during the study period
Spinazzi et al., 2007 [56]	Case study	not applicable	not applicable	Hypocupremia and hyperzincemia

**Table 3:** Summary of the impact of denture adhesives on biofilm formation.

Authors, Year	Type of Study	Change in Biofilm Formation	Time of Exposition	Dental Adhesive
Kim et al., 2003 [72]	In vivo	not significantly change	14 days	Poly Grip Free; Glaxo Smith Kline, U.K.
Oliveira et al., 2010 [73]	In vivo	no change	60 days	Ultra Corega adhesive tape (GlaxoSmithKline), Rio de Janeiro, Brazil
Makihira et al., 2001 [75]	In vitro	yeast growth	Not mentioned	six commercial denture adhesives
Rajaram et al., 2017 [76]	In vitro	antifungal	6 h, 24 h, 48 h, and 120 h	powder, cream, and strip forms of denture adhesive (Secure; Bioforce USA)
Leite et al., 2014 [77]	In vivo	growth of *Streptococcus mutans*	15 days	Ultra Corega Cream (GlaxoSmithKline Brasil Ltd.a)
Ozkan et al., 2012 [78]	In vivo	no change	2 months	(Kukident, Procter&Gamble Co., Geneva, Switzerland
Costa et al., 2022 [84]	In vitro	higher biofilm formation	24 h	ultra Corega cream, Corega strip adhesive, and ultra Corega powder (GlaxoSmithKline)
de Oliveira et al., 2018 [85]	In vitro	increased adhesion of *Candida albicans*	24 h	Ultra Corega Cream adhesive (CA) and Corega Strips adhesive (SA)
Peralta et al., 2023 [89]	In vitro	antimicrobial activity against *Candida albicans*	12 h	COREGA^®^ denture powder adhesive with Ag NPs

## Supplementary Materials

The following supporting information can be downloaded at: https://www.mdpi.com/article/10.3390/dj13110535/s1; Checklist S1: PRISMA 2020 checklist for systematic reviews.
