# Supplementary material for: Biocompatibility of Denture Adhesives: Effects on Oral Tissues and Inflammatory Responses—Literature Review
Source: Dent J (Basel). 2025 Nov 14;13(11):535. doi: 10.3390/dj13110535 (PMC12651305; doi:10.3390/dj13110535)
Supplement: Supplementary file 1 [file dentistry-13-00535-s001.zip › dentistry-3879526-supplementary.pdf]

Authors, year	Type of study	Change in biofilm formation	Time of exposition	Dental adhesive
Kim *et al.*, 2003 [75]	*In vivo*	not significantly change	14 days	Poly Grip Free; Glaxo Smith Kline, U.K.
Oliveira *et al.*, 2010 [76]	*In vivo*	no change	60 days	Ultra Corega adhesive tape (GlaxoSmithKline), Rio de Janeiro, Brasil
Makihira *et al.*, 2001 [78]	*In vitro*	yeast growth	Not mentioned	six commercial denture adhesives
Rajaram *et al.*, 2017 [79]	*In vitro*	antifungal	6 hours, 24 hours, 48 hours, and 120 hours	powder, cream, and strip forms of denture adhesive (Secure; Bioforce USA)
Leite *et al.*, 2014 [80]	*In vivo*	growth of *Streptococcus mutans*	15 days	Ultra Corega Cream (GlaxoSmithKline Brasil Ltda)
Ozkan *et al.*, 2012 [81]	*In vivo*	no change	2 months	(Kukident, Procter&Gamble Co., Geneva, Switzerland
Costa *et al.*, 2022 [87]	*In vitro*	higher biofilm formation	24 hours	ultra Corega cream, Corega strip adhesive, and ultra Corega powder (GlaxoSmithKline)
de Oliveira *et al.*, 2018 [88]	*In vitro*	increased adhesion of *Candida albicans*	24 hours	Ultra Corega Cream adhesive (CA) and Corega Strips adhesive (SA)
Peralta *et al.*, 2023 [92]	*In vitro*	antimicrobial activity against *Candida albicans*	12 hours	COREGA® denture powder adhesive with Ag NPs,
